# Editorial: Plant Secondary Compounds in Forest Ecosystems Under Global Change: From Defense to Carbon Sequestration

**DOI:** 10.3389/fpls.2019.00831

**Published:** 2019-06-26

**Authors:** Judy Simon, Bartosz Adamczyk

**Affiliations:** ^1^Plant Interactions Ecophysiology Group, Department of Biology, University of Konstanz, Konstanz, Germany; ^2^Natural Resources Institute Finland (LUKE), Helsinki, Finland

**Keywords:** climate change, boreal, temperate, eucalypts, plant secondary metabolites, herbivory, phenolics, volatiles

Forests are crucial for the future of human society, not only by their provision of fundamental ecosystems services, such as timber, food, and recreation, but also for mitigating climate change via the capture and long-term storage of carbon (MEA, 2005; Sabatini et al., 2019). At the ecosystem level, plant secondary compounds (PSCs) drive key processes, such as litter degradation, and thus nutrient cycling (Chomel et al., 2016; Adamczyk et al., 2018). At the plant level, PSCs are employed to counter herbivore attacks, cope with changes in the environment, and ultimately ensure plant survival. PSCs are chemically diverse and their abundance shifts depending on biotic and abiotic stressors, but also across species and even organs within a species. The research highlighted in this collection combines short- and long-term studies in the greenhouse and in the field with a focus on phenolics and terpenes, both at the tree and ecosystem level.

Climate change factors, such as elevated atmospheric CO_2_ levels and/or enhanced temperature might lead to counteracting responses of trees and forest ecosystems. Holopainen et al. reviewed how forest ecosystems change depending on the influence of abiotic and biotic factors on PSC synthesis. Different aspects of climate change show counteracting effects on PSCs: for example, in response to rising CO_2_ total phenolics increased and terpenes decreased in the leaves, whereas warming showed the opposite effect (Holopainen et al.). According to Mäki et al. the temporal dynamics of PSCs such as monoterpenes in forest ecosystems also depend on temperature and rainfall and are linked to the degradation of litter. Berini et al. used an untargeted metabolomics approach for five tree species and concluded that effects of the environment are species-specific and differ depending on the abiotic factors in question. However, indirect consequences of measures to mitigate global change are likely to cause further problems. For example, fertilization with nitrogen to increase carbon sequestration might result in increased insect herbivory in the future. The phenolic levels of mature *Picea abies* trees were reduced after 13 years of nitrogen deposition which—in combination with higher nitrogen content in the needles—might severely decrease tree resistance to herbivory (Nybakken et al.). In contrast, in *Picea abies*, levels of the PSCs dihydroflavonol and flavan-3-ol increased after inoculation with a fungus related to bark beetle attack and negatively affected both bark beetle and fungus (Hammerbacher et al.).

The allocation of resources to PSCs is however not just regulated by environmental factors, but also by inherent aspects. For example, the genetic variation of *Pinus pinaster* in their constitutive and induced resistance against herbivory was explained by PSC profiles rather than single compounds (López-Goldar et al.). Stark and Martz investigated whether dioecious *Juniperus communis* shows a gender specific response to a reduction in biomass because females might allocate more resources to defense (i.e., PSCs), whereas males invest more into growth. However, genders did not differ significantly in their PSC levels, most likely due to a high demand of PSCs to adapt to abiotic stresses (Stark and Martz). Moreover, Marques dos Santos et al. used a new method combining UHPLC, DAD, and MS/MS analyses to show that formylated phloroglucinols were found in glands embedded subdermally in leaves, flowers, and flower buds in eight eucalypts and one *Corymbia* species.

In conclusion, PSCs are important drivers of ecosystem processes and directly affect the interactions between trees and other organisms as well as the environment. Considering that most studies investigate tree seedlings or only the short-term effects on PSCs (Holopainen et al.), the new knowledge gained from the studies presented in this research topic is a further step in understanding the responses of trees to biotic and abiotic stressors and the underlying mechanisms at the tree and forest level, and ultimately in developing and evaluating sustainable management strategies to adapt forests to global change.

## Author Contributions

All authors listed have made a substantial, direct and intellectual contribution to the work, and approved it for publication.

### Conflict of Interest Statement

The authors declare that the research was conducted in the absence of any commercial or financial relationships that could be construed as a potential conflict of interest.
